# Parental Influence on Children’s Media Use in South Korea: National Population-Based Study

**DOI:** 10.2196/75292

**Published:** 2026-01-28

**Authors:** Ji Young Kim, Ah Jung Yang, Hye Eun Lee

**Affiliations:** 1 Department of Psychology, School of Behavioral Sciences and Education, Pennsylvania State University, Middletown, PA, United States; 2 Communication & Media Research Center, College of Social Science, Ewha Womans University, Seoul, Republic of Korea; 3 Department of Communication & Media, College of Social Science, Ewha Womans University, #403 Ewha-POSCO, 52 Ewhayeodae-gil, Seodaemun-gu, Seoul, 03760, Republic of Korea, 82 0232776937

**Keywords:** children media use, parent attitude, parent media use, parental mediation, parenting style

## Abstract

**Background:**

To better understand the effects of media use on children, it is essential to examine the various factors influencing the media use of digital native children. In the situational context, parental media usage, parents’ attitudes toward media, and parenting styles have all been identified as significant factors influencing children’s media use. This study focuses on the key factors and examines these relationships in greater depth, drawing on existing research to understand their impact on the media usage patterns of digital native children.

**Objective:**

This study examines parental influences related to young children’s media use in Korea over a 3-year period (2022‐2024) using independent, nationally representative cohorts.

**Methods:**

Using multigroup structural equation modeling, we analyzed data from 3 independent parent–reported cohorts (for 2022, n=1058; for 2023, n=1020; for 2024, n=1020) to investigate how parental media habits, attitudes, and distinct parenting styles predict children’s daytime and nighttime media consumption.

**Results:**

The online survey results revealed that parental media time, particularly for mothers, consistently correlated with higher levels of children’s daytime media use (*β*=.002-.003). Positive parental attitudes toward media increased children’s daytime media use (*β*=.028-.102), whereas negative attitudes had a limited effect (*β*=−.069-.140). Among the 7 parenting styles, positive parenting consistently reduced children’s daytime media use in 2022 and 2023 (*β*=−.228 for 2022, *β*=−.215 for 2023), but harsh punishment emerged as the strongest factor in daytime media use in 2024 (*β*=−.078 for 2022, *β*=−.090 for 2023, and *β*=−.072 for 2024). Notably, parenting styles showed no significant effect on children’s nighttime media use throughout the study, suggesting that parental influence may be more effective during daytime hours.

**Conclusions:**

This analysis extends existing research by differentiating media use patterns across time periods and highlights the evolving influence of parenting styles. These findings have implications for the development of targeted parental guidelines for managing young children’s media exposure, especially as digital media continues to become a pervasive part of daily life.

## Introduction

### Parental Influences on Children’s Media Use in South Korea

An increasing number of children access media at younger ages, with many actively consuming it. In the United States, 70% of children under the age of 1 year and 91% of children aged 2-3 years access media several times per week [1]. Similarly, in South Korea, approximately 60% of children aged 3-9 years are exposed to television before their first birthday, and 30% begin using smartphones before the age of 1 year [2]. This growing trend is in contrast with the recommendation by the American Academy of Pediatrics [3], which advises against media use for children under the age of 2 years [4]. Despite the recommendation, children’s media consumption remains high. The typical 8- to 10-year-old children spend almost 8 hours daily across different media, and this number increases for older children and teenagers to more than 11 hours per day [1].

While media may not be a leading cause of major health concerns, previous research suggests that media exposure contributes to various risks and health problems [3]. For example, younger children’s media use, as early as preschool years, has been associated with developmental difficulties such as reduced physical fitness and psychosocial health [5,6]. Specifically, engagement in risky online social behaviors [7], poor academic performance [8], obesity [9,10], reduced quality of life [11], and impaired executive function [12] have been associated with increased children’s media use. Additionally, previous studies have shown that excessive television viewing can lead to cognitive, language, and social-emotional delays [13-16]. Research has further shown that infants and toddlers gain more developmentally beneficial skills through play with physical objects rather than screen-based devices [17,18].

Numerous longitudinal [19] and cross-sectional studies [5,13,20] showed that children with greater screen time had a higher level of behavioral difficulties. A longitudinal study investigating children between the ages of 2 and 6 years showed an association between increased media use and poorer well-being outcomes, even though the results varied for boys and girls [13]. Additionally, each extra hour of television viewing per week between the ages of 2 and 4 years was associated with a 5% increase in body mass index at the age of 10 years, a 9%‐10% rise in the consumption of soda and snacks, and a 13% reduction in outdoor weekend activities [21]. A study from South Korea with survey data from mothers and teachers of 5- and 6-year-old children showed that increased use of smart devices was associated with increased levels of aggression [22]. Altogether, the findings suggest that children’s excessive consumption of media may have adverse developmental and behavioral effects.

To better understand the effects of media use on children, it is essential to examine the various factors influencing the media use of digital native children (ie, children who have access to and utilize a wide range of media and platforms from birth) [23]. One key factor is the physical environment, or in other words, the advancements in digital media technology that enable children to use media freely without restrictions of time and space through traditional means such as television as well as portable devices like smartphones and tablet PCs. Another key factor is the situational context, such as the influence of parents, given that parents shape their children’s behaviors and attitudes as primary role models [24-26]. Parental media usage [27-29], attitudes toward media [30,31], and parenting styles [26,32] have all been identified as significant factors influencing children’s media use. In addition, the impact of these factors varies according to the developmental stage or age of the children [25,26,32]. This study seeks to focus on the second key factor and examine these relationships in greater depth, drawing on existing research to understand their impact on the media usage patterns of digital native children.

Although extensive research has examined children’s overall media or screen time, much less is known about how parental factors influence media use across different times of day. This is a critical gap because daytime and nighttime media use may have distinct developmental implications (eg, school readiness, sleep quality, and behavioral regulation). Moreover, little evidence exists on how these influences shift across post-COVID years when children’s reliance on screen-based media has been both normalized and expanded to include educational contexts [33,34]. This study addresses this problem by examining parental media use, attitudes, and parenting styles as predictors of children’s daytime and nighttime media use in a large, national Korean sample drawn from three independent cohorts across 3 years (2022‐2024). In this study, “media use” is defined operationally as children’s screen-based engagement, including television, smartphones, tablets, and computers, as reported by parents in a 24-hour matrix of daily activities. Conceptually, we focus on electronic and online media rather than print or offline formats, as these account for the majority of children’s daily exposure in contemporary contexts.

### Parents’ Media Use and Social Learning Theory

Children’s screen time can be explained by Bandura’s social learning theory [35], which posits that learning and behavior are shaped by observing others in one’s environment. Young children learn by observing their parents, siblings, and others around them, picking up on daily habits, social interactions, and responses to various situations, including media use in the home. When parents model media behaviors, their children are likely to replicate those patterns. For instance, a national survey found that children often imitate their fathers or older siblings by playing with game controllers, even when they lack the skills to use them effectively [36].

Parents’ prolonged or habitual consumption of media at home may serve as a role model for children’s media use. Research indicates that parents’ media use and habitual patterns of consumption are associated with increased media use time and habitual use among children [37]. Notably, children’s media use was more closely related to their parents’ habitual consumption of media than to parental restrictions on media use. Further, studies have shown that children’s screen time tends to be higher when their mothers report substantial media use [38,39]. These findings suggest that parents’ media use may significantly influence children’s ability to regulate their own media consumption. Accordingly, we propose:

- H1: A parent’s screen time will be positively related to a child’s media use.

### Parent Attitudes and Media

Parent attitudes are another important factor in children’s screen time. Parents play a critical role in establishing rules within the household, with their influence on media consumption being particularly pronounced in younger children. Parent attitudes toward media, whether positive or negative, shape the rules governing media use, thereby directly influencing the amount of time children spend engaging with media [26]. Given their substantial influence on their children’s behaviors [40], parents often modify their behaviors as a strategy to affect changes in their children’s behaviors [41].

In the United States, a survey focused on electronic media use among children aged 6 months to 6 years indicated that parents had varied attitudes toward media use [36]. Some parents encouraged media use, finding it beneficial for children and useful for household management, such as keeping children occupied while doing chores. While similar proportions of parents felt that television had both positive (38%) and negative (31%) effects on learning, the majority supported computers as beneficial (70%) but video games as detrimental (49%) [29]. It is possible that parents aim to help their children maximize the benefits of the rich resources offered by video media while simultaneously shielding them from harmful content [42].

In general, the children of parents who view media positively tend to have higher screen time. Among preschoolers, parent attitudes and beliefs about media were strong indicators of children’s screen time [30]. For younger (0‐2 y) and older (5‐6 y) children, positive parent attitudes have been shown to significantly contribute to exceeding the recommended screen time limits set by the American Academy of Pediatrics [3,43], particularly in relation to television viewing [44]. Although parent attitudes significantly influenced the television and computer use of children, their influence on mobile devices was less pronounced [31].

Additionally, parental rules based on positive attitudes tend to support media use in the home, while negative attitudes encourage restrictions. The more critically parents evaluate media, the more likely they are to engage positively in parental mediation (active or restrictive) to mitigate the negative effects of their children’s media exposure [41]. Parental mediation has been shown to effectively influence children’s responses to media exposure [45], and experts have even advocated for increasing the level of parental mediation provided by parents [46]. Therefore, we hypothesize:

- H2: Parents with a positive attitude toward media will have children with higher levels of media use.
- H3: Parents with a negative attitude toward media will have children with lower levels of media use.

### Parenting Style and Media Use

Finally, parents considerably contribute to children’s media use, indicating that parents may mediate children’s media use and, in turn, the overall developmental outcome. For example, while children with increased media use showed a decreased level of prosocial behavior, the level of parent-child interactions mediated the association between the children’s media use and level of prosocial behavior [19]. Specifically, parenting style—or specific sets of parental mediation strategies and behaviors—may explain children’s varying levels of media use. Previous studies have shown that parental control over children’s access to smart media influences the amount of time younger children spend using such media [47]. Research in Korea has further indicated that among parental mediation strategies, supervisory control tends to be more pronounced before the age of 6 years but diminishes after the age of 7 years. In particular, for children aged 5-6 years, multiple factors—including media use patterns, self-regulatory abilities, and parental supervision—exert significant influence. This suggests that age-specific approaches to parents' mediation are necessary [48]. Research also indicates that mediation practices vary not only in form (eg, restrictive, active, or co-use) but also in effectiveness, depending on the child’s age. For younger children, restrictive mediation (eg, setting time limits) has been shown to reduce overall screen exposure, whereas older children and adolescents benefit more from active mediation approaches that involve discussion, explanation, and joint engagement [25,32,49-51].

Baumrind [52] and Maccoby and Martin [53] identified four different parenting styles: authoritative, authoritarian, indulgent, and neglectful. Each parenting style varied in their levels of demandingness and responsiveness. Generally, studies showed that children with authoritative parents (ie, highly demanding and responsive) had the most positive developmental outcome, while children with authoritarian (ie, highly demanding but not responsive) and permissive (ie, not demanding but highly responsive) parents had more negative outcomes.

The Ghent Parental Behavior Scale, developed by Leeuwen and Vermulst [54], categorizes parenting strategies into 9 distinct domains: positive parenting, autonomy, rules, monitoring, discipline, harsh punishment, material reward, inconsistent discipline, and ignoring. A material reward is a tangible incentive, such as a toy, snack, small gift, or even cash, provided to a child in exchange for a specific behavior or compliance. Positive parenting styles, such as permissive and neglectful parenting, are characterized by strategies including autonomy, positive parenting, and rules. In contrast, negative parenting styles, such as authoritative or authoritarian approaches, are associated with parenting strategies including monitoring, harsh punishment, material rewards, and ignoring [54].

A recent study by Lee et al [26] showed that a child’s daytime media use between the ages of 4 and 6 years significantly increased for parents who gave more autonomy to their children (ie, permissive and neglectful parenting style), and a child’s nighttime media use decreased with the parenting style of discipline (ie, authoritative and authoritarian parenting style). Lee et al also found that parents’ use of material rewards was the strongest predictor of the child’s nighttime media use, as the child’s nighttime media use significantly increased when parents practiced material reward. Similarly, other studies showed that children between the ages of 10 and 11 years exhibited higher levels of screen exposure when they had parents with lower levels of control over their children [27,32]. The parental behavior in this example aligns with a permissive and neglectful parenting style, where parents provide more autonomy to their children and incorporate positive parenting. On the contrary, studies showed that parents who exercise higher levels of control and support would engage in behaviors or strategies employing active and restrictive mediation [32,55]. Specific strategies would include monitoring and rules, discipline, harsh punishment, and material reward. Collectively, the literature suggests that children with permissive and neglectful parents would be more likely to have higher levels of media use, while children with authoritarian and authoritative parents would be more likely to have lower levels of media use [26].

Despite the clear implications of the parent’s role in shaping children’s media use, previous studies have focused on descriptive and correlational analyses. As Lee et al [26] suggested, further investigation on the relationship between parental determinants, such as parenting style and children’s media use, is needed. The primary goal of the study is to determine the relationship between parenting style and children’s media use across various age groups. The researcher distributed surveys to parents because young children cannot reliably self-report their daily media use, and parent reports are generally considered appropriate for estimating children’s media use, such as video and app games, computer use, and television viewing [56]. Based on previous findings that parenting style may potentially have important effects on children’s media use, we hypothesize the following:

- H4: Specific parenting styles will be associated with children’s level of media use.

The secondary goal of the study is to extend the findings of Lee et al [26] by replicating the previous findings. Therefore, we incorporated the 2 hypotheses related to parenting style used in Lee et al [26]:

- H5: Children who have parents with permissive and neglectful parenting styles (ie, exercise positive parenting and give autonomy to children) will have higher levels of media use.
- H6: Children who have parents with authoritative and authoritarian parenting styles (ie, monitoring, rules, discipline, harsh punishment, and material reward) will have lower levels of media use.

Previous findings support a clear relationship between parenting style and children’s media use. To extend the existing literature, it is imperative to identify the parental determinant that explains children’s media use, and the researcher of this study aims to answer the following research question:

- RQ1: Among parent’s media use, media attitudes, and parenting styles, what is the most influential factor on children’s media use?

## Methods

### Participants

A total of 1058 parents of children aged 5-7 years, 1020 parents of children aged 6-8 years, and 1020 parents of children aged 7-9 years completed questionnaires between April 26 and May 9, 2022; October 26 and November 9, 2023; and July 31 and August 21, 2024, respectively. All participants completed the questionnaire through an online survey conducted by a Korean survey company, Macromill Embrain, which recruited participants from its national panel pool. Each year’s sample primarily consisted of an independent cross-sectional group, although approximately 5%‐10% of the participants may have overlapped across years due to the nature of the panel system. However, the study did not track individuals over time, and analyses were conducted separately for each dataset. The age and sex distribution of the children was balanced across each dataset. Detailed demographic characteristics for all three groups are presented in Table 1.

**Table 1.** Demographic characteristics of participants and their child (age, gender, education level, income, employment, and family members).

Year and demographic characteristics	Participants, n (%)
2022	
Age (child) (y)	
5	359 (33.9)
6	352 (33.3)
7	347 (32.8)
Gender (child)	
Male	535 (50.6)
Female	523 (49.4)
Age (parent) (y)	
20	15 (1.4)
30	578 (54.6)
40	453 (42.8)
50	12 (1.1)
Gender (parent)	
Male	129 (12.2)
Female	929 (87.8)
Family members	
2	11 (1.0)
3	301 (28.4)
4	576 (54.4)
5	109 (10.3)
>6	61 (5.8)
Education level (father)	
High school or below	144 (13.6)
College or university	781 (73.8)
Master’s degree or above	133 (12.6)
Education level (mother)	
High school or below	108 (10.2)
College or university	834 (58.8)
Master’s degree or above	116 (11.0)
Monthly income (family) (USD)	
<4000	341 (32.2)
4000‐5000	235 (22.2)
5000‐6000	161 (15.2)
>6000	321 (30.3)
Employment (father)	
Full-time job	993 (93.9)
Part-time job	50 (4.7)
No job	15 (1.4)
Employment (mother)	
Full-time job	519 (49.1)
Part-time job	171 (16.2)
No job	368 (34.8)
Total	1058 (100)
2023	
Age (child) (y)	
6	340 (33.3)
7	340 (33.3)
8	340 (33.3)
Gender (child)	
Male	510 (50.0)
Female	510 (50.0)
Age (parent) (y)	
20	5 (0.5)
30	468 (45.9)
40	541 (53.0)
50	6 (0.6)
Gender (parent)	
Male	144 (14.1)
Female	876 (85.9)
Family members	
2	317 (31.1)
3	547 (53.6)
4	105 (10.3)
5	31 (3.0)
>6	20 (2.0)
Education level (father)	
High school or below	128 (12.6)
College or university	759 (74.4)
Master’s degree or above	133 (13.0)
Education level (mother)	
High school or below	90 (8.8)
College or university	803 (78.7)
Master’s degree or above	127 (12.5)
Monthly income (family) (USD)	
<4000	205 (20.1)
4000‐5000	222 (21.8)
5000‐6000	173 (16.9)
>6000	420 (41.2)
Employment (father)	
Full-time job	968 (94.9)
Part-time job	41 (4.0)
No job	11 (1.1)
Employment (mother)	
Full-time job	576 (56.5)
Part-time job	160 (15.7)
No job	284 (27.8)
Total	1020 (100)
2024	
Age (child) (y)	
7	340 (33.3)
8	340 (33.3)
9	340 (33.3)
Gender (child)	
Male	510 (50.0)
Female	510 (50.0)
Age (parent) (y)	
20	7 (0.7)
30	369 (36.2)
40	628 (61.6)
50	16 (1.6)
Gender (parent)	
Male	133 (13.0)
Female	887 (87.0)
Family members	
2	307 (31.1)
3	549 (53.8)
4	119 (11.7)
5	33 (3.2)
>6	12 (1.2)
Education level (father)	
High school or below	123 (12.1)
College or university	741 (72.6)
Master’s degree or above	156 (15.3)
Education level (mother)	
High school or below	82 (8.0)
College or university	810 (79.5)
Master’s degree or above	128 (12.5)
Monthly income (family) (USD)	
<4000	172 (16.9)
4000‐5000	197 (19.3)
5000‐6000	179 (17.5)
>6000	472 (46.3)
Employment (father)	
Full-time job	976 (95.7)
Part-time job	40 (3.9)
No job	4 (0.4)
Employment (mother)	
Full-time job	602 (59.0)
Part-time job	153 (15.0)
No job	265 (26.0)
Total	1020 (100)

### Ethical Considerations

The study received ethical approval from the Institutional Review Board of Ewha Womans University (Institutional Review Board approval number: EWHA-202103-0028-01). All data were anonymized prior to analysis, and no personally identifiable information was stored or retained. Informed consent was obtained from all participants involved in the study. As the participants were members of a registered survey company panel, an honorarium was provided based on the survey response time at a rate of 100 KRW (approximately US $0.67) per minute. Participants who discontinued the survey prior to completion were also compensated with a prorated stipend corresponding to their participation time.

### Instrument and Measures

#### Overview

The questionnaire was initially developed in English and then translated into Korean, with translation equivalence verified by bilingual researchers. In addition to the main variables, media use (ie, time spent on media via television, personal device, smartphone) and demographic information of both children and their parents were collected. Tables 2, 3, 4 present the reliabilities and descriptive statistics of the variables, along with their correlations. Composite variables were calculated once unidimensionality and acceptable reliability were confirmed. All variables were measured with 5-point Likert scales (1=“strongly disagree” to 5=“strongly agree”) unless otherwise noted.

**Table 2.** Reliabilities, correlations, means, and standard deviations of the main variables (2022)^a^.

2022	1	2	3	4	5	6	7	8	9	10	11	12	13	14	15
Child’s age at first use	—^b^														
Child’s locus of control	–0.05	(0.75)													
Mother’s media time	0.01	0.17^c^	—												
Father’s media time	0.04	0.13^c^	0.62^c^	—											
Positive attitude toward media use	0.10^c^	–0.02	0.10^c^	0.05	(0.90)										
Negative attitude toward media use	0.01	0.44^c^	0.03	0.07^d^	–0.26^c^	(0.89)									
Positive parenting	–0.05	–0.19^c^	–0.06	–0.03	–0.09^c^	–0.12^c^	(0.89)								
Monitoring	0.01	–0.06^d^	–0.07^d^	–0.02	0.10^c^	0.02	0.48^c^	(0.72)							
Rules	–0.07^d^	–0.11^c^	0.01	0.02	0.01	–0.01	0.52^c^	0.44^c^	(0.84)						
Discipline	0.00	0.13^c^	0.02	0.04	0.05	0.21^c^	–0.05	0.10^c^	0.21^c^	(0.76)					
Harsh punishment	0.05	0.26^c^	0.11^c^	0.07^d^	0.10^c^	0.22^c^	–0.36^c^	–0.16^c^	–0.20^c^	0.34^c^	(0.92)				
Material reward	0.05	0.25^c^	0.11^c^	0.10^c^	0.21^c^	0.12^c^	–0.04	0.07^d^	0.00	0.21^c^	0.25^c^	(0.76)			
Autonomy	0.05	–0.10^c^	0.01	0.06	0.13^c^	–0.03	0.45^c^	0.33^c^	0.37^c^	0.03	–0.13	0.07^d^	(0.80)		
Child’s daytime media use	–0.08^d^	0.34^c^	0.26^c^	0.17^c^	0.04	0.10^c^	–0.12^c^	–0.07^d^	–0.06	0.05	0.05	0.08^c^	–0.04		—
Child’s nighttime media use	–0.01	0.12^d^	0.12^c^	0.11^c^	–0.05	0.02	–0.05	–0.06	–0.04	0.05	0.08^c^	0.01	–0.03	0.09**^c^	
Mean (SD; range)	3.33 (1.36; 1‐8)	2.88 (0.82; 1‐5)	120.67 (83.81; 0‐600)	127.10 (86.03; 0‐600)	2.92 (0.70; 1‐5)	3.09 (0.68; 1‐5)	4.01 (0.47; 1.91‐5)	3.79 (0.60; 1‐5)	4.12 (0.46; 1.86‐5)	3.31 (0.66; 1‐5)	1.71 (0.86; 1‐4.75)	2.83 (0.84; 1‐5)	3.83 (0.53; 2‐5)	2.37 (1.49; 0‐13)	0.19 (0.48; 0‐4.29)

a Reliabilities, calculated using Cronbach α, are reported in parentheses on the diagonal.

b Not applicable.

c *P*<.001.

d *P*<.05.

**Table 3.** Reliabilities, correlations, means, and standard deviations of the main variables (2023)^a^.

2023	1	2	3	4	5	6	7	8	9	10	11	12	13	14	15
Child’s age at first use	—^b^														
Child’s locus of control	–0.04	(0.76)													
Mother’s media time	0.01	0.16^c^	—												
Father’s media time	–0.03	0.15^c^	0.59^c^	—											
Positive attitude toward media use	0.11^c^	–0.05	–0.02	–0.08^c^	(0.92)										
Negative attitude toward media use	–0.01	0.42	0.02	0.05	–0.22^c^	(0.90)									
Positive parenting	–0.03	–0.22^c^	–0.13^c^	–0.07^d^	0.15^c^	–0.11^c^	(0.9)								
Monitoring	–0.03	–0.05	–0.05	0.01	0.08^c^	0.05	0.50^c^	(0.69)							
Rules	–0.07^d^	–0.11^c^	–0.02	0.02	0.01	–0.01	0.54^c^	0.49^c^	(0.85)						
Discipline	–0.04	0.13^d^	0.02	0.04	0.07^d^	0.13^c^	0.04	0.23^c^	0.26^c^	(0.74)					
Harsh punishment	0.06	0.25^c^	0.12^c^	0.09^c^	0.16^c^	0.13^c^	–0.35^c^	–0.16^c^	–0.25^c^	0.25^c^	(0.92)				
Material reward	0.06	0.26^c^	0.07^d^	0.03	0.13^c^	0.16^c^	–0.08^d^	–0.00	–0.06^d^	0.17^c^	0.25^c^	(0.77)			
Autonomy	0.01	–0.13^c^	–0.04	–0.03	0.07^d^	–0.05	0.47^c^	0.31^c^	0.38^c^	0.11^c^	–0.15^c^	0.02	(0.81)		
Child’s daytime media use	–0.10^c^	0.32^c^	0.25^c^	0.22^c^	–0.05	0.10^c^	–0.10^c^	–0.05	–0.01	0.04	0.02	0.05	–0.03		—
Child’s nighttime media use	–0.01	0.15^c^	0.11^c^	0.10^c^	–0.03	0.04	–0.08	–0.04	–0.04	0.06	0.05	0.07^d^	–0.01	0.16^c^	
Mean (SD; range)	3.46 (1.50; 1‐9)	2.83 (0.84; 1‐5)	117.53 (81.51; 0‐610)	125.65 (83.92; 0‐600)	2.85 (0.72; 1‐4.89)	3.16 (0.67; 1‐5)	4.04 (0.48; 2.09‐5)	3.90 (0.57; 1.75‐5)	4.32 (0.50; 2.33‐5)	3.50 (0.72; 1‐5)	1.70 (0.83; 1‐4.75)	2.79 (0.83; 1‐5)	3.86 (0.52; 2‐5)	2.35 (1.48; 0‐10.71)	0.22 (0.46; 0‐4)

a Reliabilities, calculated using Cronbach α, are reported in parentheses on the diagonal.

b Not applicable.

c *P*<.001.

d *P*<.05.

**Table 4.** Reliabilities, correlations, means, and standard deviations of the main variables (2024)^a^.

2024	1	2	3	4	5	6	7	8	9	10	11	12	13	14	15
Child’s age at first use	—^b^														
Child’s locus of control	–0.06	(0.76)													
Mother’s media time	0.02	0.13^c^	—												
Father’s media time	–0.06^d^	0.08^c^	0.54^c^	—											
Positive attitude toward media use	0.04	0.02	0.04	–0.03	(0.93)										
Negative attitude toward media use	–0.01	0.23^c^	0.01	0.01	–0.32^c^	(0.90)									
Positive parenting	–0.01	–0.11^c^	–0.11^c^	–0.07^d^	0.08^d^	–0.08^d^	(0.87)								
Monitoring	–0.02	–0.04	–0.02	0.02	0.03	0.05	0.47	(0.72)							
Rules	–0.05	–0.06	–0.02	0.04	–0.08^d^	0.03	0.54	0.45^c^	(0.92)						
Discipline	–0.02	0.10^c^	0.07^d^	0.06^d^	0.07^d^	0.18^c^	0.06	0.15^c^	0.14^c^	(0.72)					
Harsh punishment	0.02	0.11^c^	0.12^c^	0.09^c^	0.14^c^	0.12^c^	–0.29^c^	–0.14^c^	–0.24^c^	0.37^c^	(0.92)				
Material reward	0.04	0.15^c^	0.03	0.02	0.17^c^	0.14^c^	–0.17^c^	0.01	–0.14^c^	0.23^c^	0.37^c^	(0.77)			
Autonomy	0.04	–0.04	–0.06	–0.04	0.06	0.02	0.40^c^	0.22^c^	0.32^c^	0.01	–0.11	0.01	(0.80)		
Child’s daytime media use	–0.13^c^	–0.23^c^	0.18^c^	0.14^c^	–0.05	0.05	–0.05	–0.05	–0.01	–0.02	0.02	0.02	0.03	–0.02	—
Child’s nighttime media use	–0.08^d^	0.13^c^	0.15^c^	0.13^c^	0.04	0.01	–0.01	–0.01	0.00	0.04	0.02	0.02	0.06^d^	0.02	0.23^c^
Mean (SD; range)	3.73 (1.69; 1‐10)	3.01 (0.61; 1‐5)	124.52 (85.88; 0‐600)	140.77 (87.96; 0‐600)	2.54 (0.79; 1‐4.89)	3.32 (0.67; 1‐5)	4.03 (0.46; 2.27‐5)	3.94 (0.57; 1.75‐5)	4.43 (0.52; 2.33‐5)	3.21 (0.60; 1‐5)	1.73 (0.87; 1‐5)	2.60 (0.85; 1‐5)	3.87 (0.57; 1‐5)	3.48 (2.50; 0‐14)	0.34 (0.74; 0‐10)

a Reliabilities, calculated using Cronbach α, are reported in parentheses on the diagonal.

b Not applicable.

c *P*<.001.

d *P*<.05.

#### Children’s Age at First Media Use

Parents were asked about their child’s age at first media exposure with the question, “How old was your child when he/she first started watching media content?” The response options included “less than 12 months,” “1 year,” “2 years,” “3 years,” “4 years,” “5 years,” “6 years,” and “7 years.”

#### Parents’ Media Time

Each participant reported both their own and their spouse’s media watching time. Two questions were used: “How many hours and minutes do you spend watching media on a typical weekday?” and “How many hours and minutes do you spend to watch media on a typical weekend?” Responses were averaged after weighing (ie, (weekday time × 5 days + weekend × 2 days)/7)) and converted to minutes.

#### Parents’ Positive and Negative Attitudes Toward Media Use

Based on scales by Elias and Sulkin [57] and Nikken and Jansz [58], 9 items measured positive attitudes (eg, “I think watching media will positively influence my child’s behavioral development.”). Negative attitudes were measured with 2 dimensions: intellectual and social. Each dimension included 4 items, such as “I think watching media will hurt my child’s creativity.” for the intellectual dimension and “I believe watching media will negatively affect my child’s play with friends.” for the social dimension.

#### Parenting Styles

The Ghent Parental Behavior Scale [54] was used to measure the parenting styles. This scale has demonstrated valid structure in diverse samples. Seven dimensions were included due to reliability concerns with 2 original dimensions (inconsistent discipline and ignoring). The included dimensions are positive parenting (11 items: eg, “I make time to listen to my child when he/she wants to tell me something.”), monitoring (4 items: eg, “I keep track of the friends my child is seeing.”), rules (6 items: eg, “I teach my child to obey rules.”), discipline (4 items: eg, “When my child does something I don’t approve of, I punish him/her.”), harsh punishment (4 items: eg, “I spank my child when he/she is disobedient.”), material reward (3 items: eg, “I reward my child with money or a small gift for good behavior.”), and autonomy (3 items: eg, “I teach my child to solve problems independently.”). Items excluded for reliabilities included 1 (“I teach my child respect for the authorities.”) from the rules dimension and 2 items (“When my child has been misbehaving, I give him/her a chore for punishment.” and “It happens that I don’t punish my child after he/she has done something that is not allowed.”) from harsh punishment dimension. All items and dimensions can be referenced in Leeuwen and Vermulst’s measurement study [54].

#### Child’s Media Time

Participants selected a cell in a 24-hour matrix to indicate when a child watched media during both weekdays and weekends. Daytime use was defined as viewing between 7:00 AM and 9:00 PM, while viewing before 7:00 AM or after 9:00 PM was classified as nighttime use. This classification was based on developmental sleep guidelines: given that elementary schools in Korea typically begin at 9:00 AM, and the American Academy of Sleep Medicine [59] recommends 10‐13 hours of sleep for children aged 3‐5 and 9‐12 hours for children aged 6‐12, we estimated that 10‐11 hours of sleep starting from 9:00 PM would allow children to function healthily on a daily basis. In practical terms, a 9:00 PM bedtime enables children to sleep until approximately 7:00-8:00 AM, providing sufficient time to prepare for and arrive at school. In addition, “screen time” was measured during 2022‐2024, when academic activities had resumed their normal patterns following the COVID-19 pandemic, thereby minimizing the potential impact of external variables. Media time was averaged and reported in hours. Due to the varied and skewed nature of the data, standardized *z* scores were used for analysis.

## Results

Multigroup structural equation modeling was conducted to test the hypotheses and research question using Mplus 8.0 [60], which uses the maximum likelihood estimation method. To evaluate the model fit, confirmatory fit index (CFI), goodness-of-fit index (GFI), and normed fit index (NFI) were used.

**Figure 1.**
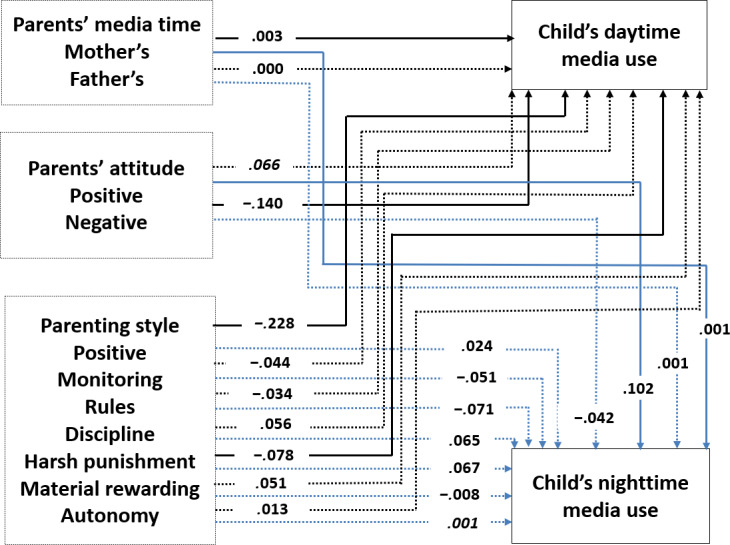
The final model of the relationships among a child’s daytime and nighttime media use, parents’ media time, parents’ attitude toward media use, and parenting style in 2022. Note that the values are the observed standardized path coefficients. Solid lines indicate significant coefficients at *P*<.05, whereas dotted lines represent nonsignificant coefficients. In addition, paths leading to nighttime media use are shown in light blue, while paths leading to daytime media use are shown in black.

Acceptable goodness-of-fit indices were obtained for the overall model [61]: *χ*^2^_1_=1.2, *P*=.27, CFI=0.99, GFI=1.00, NFI=0.99 for 2022, *χ*^2^_1_=8.9, *P*<.01, CFI=0.99, GFI=1.00, NFI=0.99 for 2023, and *χ*^2^_1_=44.7, *P*<.001, CFI=0.98, GFI=0.99, NFI=0.98 for 2024. The estimated coefficients are presented in Figures1^3^.

**Figure 2.**
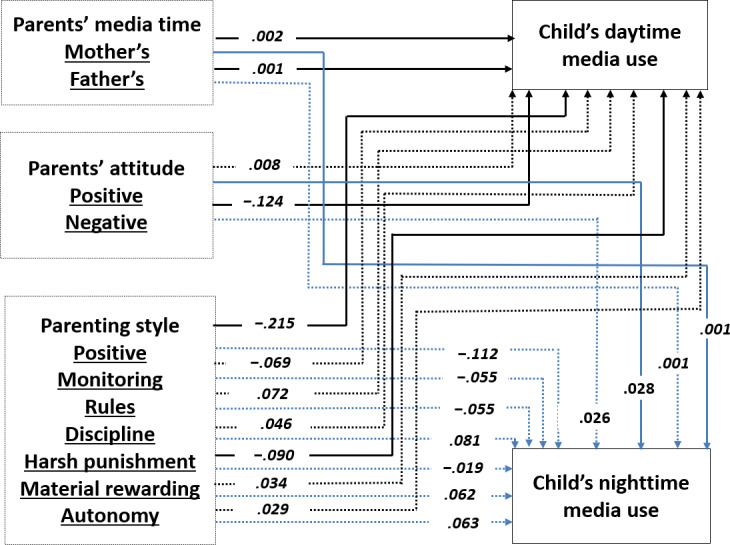
The final model of the relationships among a child’s daytime and nighttime media use, parents’ media time, parents’ attitude towards media use, and parenting style in 2023. Note that the values are the observed standardized path coefficients. Solid lines indicate significant coefficients at *P*<.05, whereas dotted lines represent non-significant coefficients. In addition, paths leading to nighttime media use are shown in light blue, while paths leading to daytime media use are shown in black.

**Figure 3.**
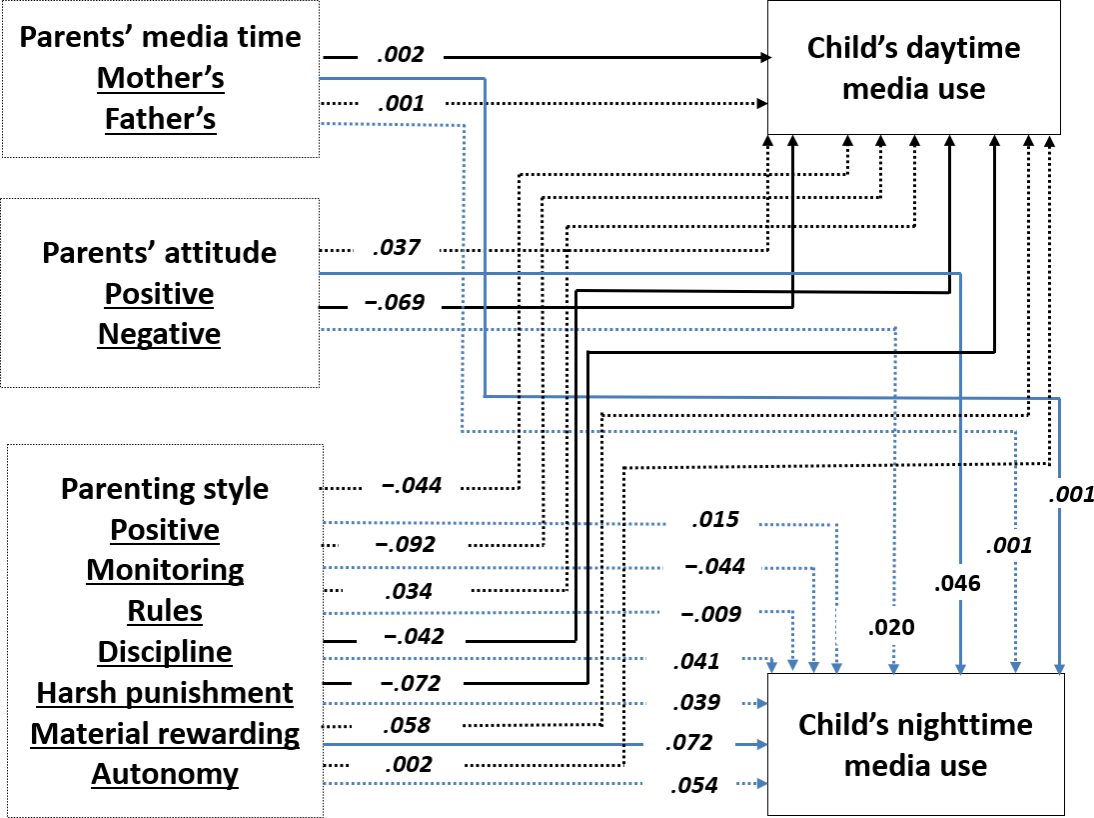
The final model of the relationships among a child’s daytime and nighttime media use, parents’ media time, parents’ attitude toward media use, and parenting style in 2024. Note that the values are the observed standardized path coefficients. Solid lines indicate significant coefficients at *P*<.05, whereas dotted lines represent nonsignificant coefficients. In addition, paths leading to nighttime media use are shown in light blue, while paths leading to daytime media use are shown in black.

H1 predicted that parents with a higher level of screen time would have children who have a higher level of media use. The results partially supported this hypothesis. Regarding a child’s daytime media use, mother’s media time had a positive effect consistently in 2022, 2023, and 2024 (*β*=.002-.003), but father’s media time showed a significant positive effect on child’s daytime in 2023 only (*β*=.001). Regarding a child’s nighttime media use, mother’s media time showed a positive effect in 2022, 2023, and 2024 (*β*=.001).

H2 and H3 hypothesized that parents’ positive and/or negative attitudes toward media use would affect child’s media use. Parents’ positive attitude toward media use increased a child’s nighttime media use (*β*=.028-.102) and parents’ negative attitude decreased a child’s daytime media use (*β*=−.069-.140). H2 and H3 were supported.

Finally, H4 predicted that parents with permissive and neglectful parenting styles would be positively associated with children’s media use, while parents with authoritative and authoritarian parenting styles would be negatively related to child’s media use. Positive parenting style decreased a child’s daytime media use (*β*=−.228 for 2022, *β*=−.215 for 2023), and harsh punishment (*β*=−.078 for 2022, *β*=−.090 for 2023, and *β*=−.072 for 2024) among the 7 parenting styles significantly decreased a child’s daytime media use. Material reward was positively associated with child’s nighttime media use (*β*=.072) in 2024.

RQ1 examined which factors were the most influential among parents’ media use, attitude on media, and parenting styles. The results showed that parents’ positive parenting style was the strongest predictor of a child’s daytime media use in 2022 and 2023, but parents’ harsh punishment was the strongest predictor in 2024. Parents’ positive and negative attitudes on media use were the strongest predictor of a child’s nighttime media use in 2022 only.

## Discussion

This study investigated the connection between young children’s media use and various parental influences, including media time, attitudes toward media, and parenting styles. The results across 2022, 2023, and 2024 consistently demonstrate the critical role parents play in shaping their children’s media use habits. Similar to previous studies, it was observed that when parents—especially mothers—engage in higher media use, their children’s media consumption during both daytime and nighttime increases, supporting the findings from past research [26,38,39]. This aligns with the idea that parental behavior directly influences children’s media use habits.

Prior research has typically collapsed media consumption into a single metric, overlooking potential temporal differences. Our findings show that parental influences such as parent attitudes and parenting styles are differentially affected by the temporal contexts (ie, daytime vs nighttime). Specifically looking at parent attitudes, across the 3 years, a negative parent attitude toward media consistently correlated with decreased daytime media use, but a positive parent attitude toward media consistently correlated with increased nighttime media use. These consistent relationships across 3 years reflect the findings by Cingel and Krcmar [30] that emphasize the predictive power of parental attitudes on children’s screen time.

Parenting style showed distinct influences on children’s media use patterns over the 3-year period. In 2022 and 2023, positive parenting emerged as a significant factor in reducing children’s daytime media use, highlighting that nurturing and supportive approaches were effective in moderating screen time during the day. By 2024, however, harsh punishment had become the strongest predictor of reduced daytime media use, suggesting a shift where more stringent disciplinary actions had a notable impact on limiting screen time. This change may reflect an evolving role of disciplinary measures in influencing children’s media habits over time.

Contrary to expectations, none of the parenting styles showed a significant association with children’s nighttime media use across the 3 years except 1. In 2024, material reward was positively associated with a child’s nighttime media use. The findings indicated that parenting styles, while impactful on daytime media consumption, may have a limited role in controlling screen time during nighttime hours. Overall, the findings of daytime and nighttime media use suggest that while positive parenting consistently influences daytime media use, the emergence of harsh punishment and material reward as a stronger factor in 2024 points to potential shifts in the effectiveness of various parenting strategies over time.

The findings from a study focused on a younger age group (aged 3-6 y) than this study [26] demonstrated that children’s daytime media use increased when parents provided more autonomy to their children. Conversely, nighttime media use decreased among children of parents who emphasized discipline. Children’s nighttime media use was significantly increased when parents employed material rewards as a motivational tool. This study extended the findings of Lee et al [26] by investigating an older group of children (ie, aged 5-7 y). The findings from the 2 studies collectively suggest that as children grow older, the effectiveness of these parental interventions, particularly in reducing nighttime media use, diminishes significantly. These findings contribute to a broader understanding of the role of parental determinants in shaping children’s media use and underscore the necessity for age-specific parental guidelines.

Previous results consistently highlighted the value of parental mediation in amplifying the positive effects and mitigating the adverse effects of children’s media exposure [45,62]. These findings have encouraged scholars to highlight the critical role of parental engagement in managing children’s media use [41]. However, a significant gap persists in the implementation of parental controls. For instance, most American adolescents report having no rules or restrictions from their parents regarding either the type of television content or the amount of time spent viewing [1].

Parents play a crucial role in ensuring safe and beneficial use of digital devices, particularly given that children’s cognitive and functional abilities are still in developmental stages. Studies have shown that parents can actively promote children’s growth and development through well-informed media management practices. Rather than relying on restrictive approaches, Wu et al [63] proposed integrating restrictive, instructive, and joint approach strategies to create a balanced and effective framework. This combined approach encourages collaboration and communication between parents and children, facilitating the establishment of developmentally appropriate media use habits that support overall growth.

Furthermore, these results extend previous research on parental determinants of children’s media use and underscore the importance of establishing specific parental guidelines tailored to different times of day. The differentiation between daytime and nighttime media use is particularly relevant, as studies have shown that media use affects multiple developmental domains, including sleep quality, cognitive development, language, and socioemotional and physical health [13-16]. The variations observed over the years suggest that a longitudinal approach can provide a more comprehensive understanding of parental influences on media habits.

From a policy perspective, these findings support the creation of targeted recommendations that help parents foster healthy media use patterns at home. With children increasingly exposed to media, guidelines that encourage informed parental choices could mitigate the potential risks associated with excessive screen time. This evidence-based approach can empower parents to optimize on- and off-screen activities for their children’s development.

However, this study is not without limitations. First, it is important to acknowledge that the data were collected during COVID-19 and post-COVID years (2022‐2024), when screen time became both more prevalent and more socially normalized. In this period, children’s daytime media use may not solely reflect leisure activities but may also encompass school-related or educational screen exposure. Second, parental self-reports remain susceptible to social desirability bias, especially given the awareness of recommended screen time limits [64]. Future research should incorporate observational methods to provide a more objective measure of media use. Additionally, distinguishing between types of media would allow for a more nuanced understanding of the effects of specific content on children’s behavior and development, as different media types (eg, gaming vs educational content) may have distinct impacts. Similarly, a limitation of this study is the reliance on parent-reported 24-hour matrix data, which, while useful for distinguishing daytime and nighttime use, does not capture the content, context, or quality of children’s media exposure. Prior work highlights that screen time measures often overlook these dimensions and that more comprehensive approaches are needed to assess family media environments [65,66]. Future studies should integrate both time-based metrics and contextual measures to provide a fuller understanding of children’s media use. Although the survey company’s online panel is designed to mirror the Korean population in terms of key demographic characteristics (eg, sex, age, and family structure), the sampling method is not entirely random. Therefore, the findings should not be interpreted as being representative of the whole population. Rather, the results should be considered in the context of the study’s sample, and caution should be exercised when generalizing these patterns to all Korean parents and children. Finally, the correlational nature of this study does not imply causation; thus, further research should explore potential genetic and environmental moderators that could influence the relationship between parental behavior and children’s media habits.

In conclusion, the study across 3 years underscores those parental influences, including media time, attitudes, and specific parenting strategies, continue to shape children’s media behaviors. By accounting for temporal patterns, researchers and policymakers can provide parents with tailored guidance to support balanced media use, enhancing children’s overall well-being in the digital age.

## Abbreviations

CFI: confirmatory fit index
GFI: goodness-of-fit index
NFI: normed fit index
